# A systematic review of the role of the nociceptin receptor system in stress, cognition, and reward: relevance to schizophrenia

**DOI:** 10.1038/s41398-017-0080-8

**Published:** 2018-02-02

**Authors:** Muhammad Saad Khan, Isabelle Boileau, Nathan Kolla, Romina Mizrahi

**Affiliations:** 1 Research Imaging Centre, Centre for Addiction and Mental Health, 250 College St., Toronto, ON M5T 1R8 Canada; 2 Institute of Medical Science, Faculty of Medicine, University of Toronto, 1 King’s College Circle, Toronto, ON M5S 1A8 Canada; 3 Campbell Family Mental Health Research Institute, Centre for Addiction and Mental Health, 250 College St., Toronto, ON M5T 1R8 Canada; 4 Department of Psychiatry, University of Toronto, 250 College St., Toronto, ON M5T 1R8 Canada

## Abstract

Schizophrenia is a debilitating neuropsychiatric illness that is characterized by positive, negative, and cognitive symptoms. Research over the past two decades suggests that the nociceptin receptor system may be involved in domains affected in schizophrenia, based on evidence aligning it with hallmark features of the disorder. First, aberrant glutamatergic and striatal dopaminergic function are associated with psychotic symptoms, and the nociceptin receptor system has been shown to regulate dopamine and glutamate transmission. Second, stress is a critical risk factor for first break and relapse in schizophrenia, and evidence suggests that the nociceptin receptor system is also directly involved in stress modulation. Third, cognitive deficits are prevalent in schizophrenia, and the nociceptin receptor system has significant impact on learning and working memory. Last, reward processing is disrupted in schizophrenia, and nociceptin signaling has been shown to regulate reward cue salience. These findings provide the foundation for the involvement of the nociceptin receptor system in the pathophysiology of schizophrenia and outline the need for future research into this system.

## Introduction

Schizophrenia is a debilitating disorder characterized by positive symptoms, such as delusions and hallucinations, and negative symptoms, such as a flat affect, alogia, and anhedonia, as well as deficits in cognition and reward modulation^1–3^. Some of the psychopathology of schizophrenia is characterized by dopaminergic and glutamatergic dysregulation, increased stress vulnerability via hypothalamic–pituitary–adrenal (HPA) axis dysregulation, cognitive deficits, which include alterations in the cholinergic system, and deficits in reward modulation^2–7^.

The nociceptin receptor (NOPr) is a G protein-coupled receptor identified in 1994, and was initially classified as a member of the opioid receptor family based on structural homology to the opioid receptors^8^. However, it was later reclassified as a non-opioid member of the opioid system, because endogenous ligands for other opioid receptors, such as the mu, kappa, and delta receptors, showed little affinity for it. The endogenous peptide, now known as nociceptin/orphanin FQ (N/OFQ), was identified in 1995, and is a heptadecapeptide with pro-nociceptive properties^9,10^. In vitro receptor autoradiography in rats and post-mortem studies in humans have shown NOPr to be widely distributed, with greater density in cortical regions and the human striatum^11,12^. Positron emission tomography (PET) studies using the ligand [^11^C]NOP-1A have corroborated these findings in vivo, with high concentrations of NOPr observed in the cerebral cortex and the striatum^13^. Given this widespread expression, it is well positioned to interact with multiple receptor systems in the brain and be involved in several functions.

In investigating the NOPr system, the majority of the literature is a result of preclinical work. This highlights a need for more research into this system as it could have potential in elucidating and treating psychiatric disorders. With regard to schizophrenia, the NOPr system may indeed have an impact given its involvement in important neurotransmitter systems and symptom clusters particularly relevant for the disorder.

## Methods

**Fig. 1:**
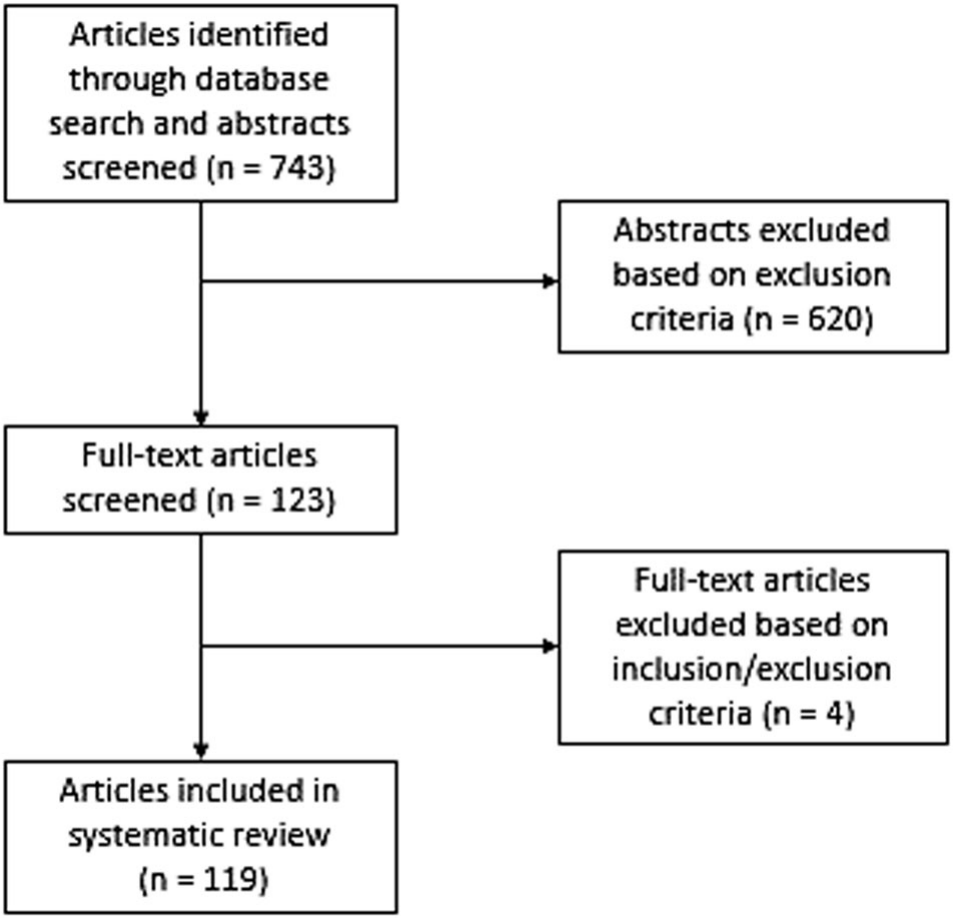
Flowchart of the searching process for articles

The goal of this systematic review is to describe the role of the NOPr system in specific systems and symptoms that are relevant to schizophrenia. Thus, in this review we searched for the involvement of the NOPr system in each of the aforementioned domains relevant to schizophrenia. A search was conducted on the MEDLINE database for all research articles from 1994 onward using the Boolean string “(nociceptin receptor OR orl1 OR N/OFQ OR nociceptin/orphanin FQ OR orphanin FQ) AND (stress OR hypothalamic–pituitary–adrenal OR HPA OR immune system OR immune cells OR cytokines OR reward OR place preference OR cognition OR learning OR memory OR acetylcholine OR potentiation OR dopamine OR glutamate OR behavior OR locomotor OR schizophrenia OR psychosis OR post mortem OR microdialysis)”. The most recent search was conducted on October 3rd 2017. The abstracts for each of the articles in the search results were then screened using the following inclusion criteria: (a) studies investigating NOPr system involvement in cognition, stress, reward, cholinergic modulation, dopamine modulation, or glutamate modulation, and (b) that could be related to the symptoms underlying schizophrenia. Exclusion criteria were as follows: (a) studies that investigated novel ligands for NOPr investigation; (b) studies investigating pain or pain mechanisms; (c) studies that were unrelated to any of the four domains mentioned above or that could not be related to schizophrenia symptomatology; and (e) general review articles. The flowchart for this process is depicted in Fig. 1. We also want to note that, while there is evidence for NOPr system involvement in serotonin, β-endorphin, and norepinephrine signaling, these were excluded from this review in favor of focus on well-supported systems in psychosis.

## Results

A total of 743 articles were obtained through the search, which were then screened. Following screening of abstracts, 123 articles were identified as potentially relevant to this review, and of these, 119 were included in this systematic review. Our discussion will thus focus on results from four areas of research relevant to psychosis: (1) NOPr system involvement in dopamine and glutamate transmission; (2) NOPr system involvement in stress and HPA modulation; (3) NOPr system involvement in cognition; and (4) NOPr system involvement in reward modulation.

### Involvement of the NOPr system in dopamine transmission

A common pathology in schizophrenia is characterized by the dopamine hypothesis, which suggests an increased striatal dopamine transmission in these individuals^4^. Evidence suggests that the NOPr system could play a role. An initial study by Norton et al. revealed the presence of NOPr on cell bodies of dopamine neurons in the midbrain and a co-localization of NOP mRNA with tyrosine hydroxylase (TH) neurons, with mRNA also present on tegmental and nigral dopaminergic neurons^14^. This localization of NOP mRNA was later confirmed with an experiment using 6-hydroxydopamine lesions in rats, in which a large loss of TH neurons led to a reduction of N/OFQ and NOPr mRNA in the caudate putamen^15^. TH is an enzyme involved in the synthesis of the dopamine precursor L-DOPA, which plays a critical role in dopamine synthesis^16^. Olianas et al. furthered these findings by demonstrating an inhibitory effect of N/OFQ on TH phosphorylation, which inhibited dopamine transmission presynaptically^17^. They also observed a selective post-synaptic downregulation of dopamine D1 receptor signaling in the nucleus accumbens and striatum after N/OFQ administration. An involvement with dopamine D2 receptor signaling is also noted given that administration of a D2 antagonist prevented the improvements in motor performance with NOPr antagonists^18^. In the same study, genetic knockout of the D2 receptor erased the motor facilitating effect of a low dose of N/OFQ, indicating that the NOPr system could also exert effects on dopamine transmission through this receptor, potentially via a presynaptic mechanism as suggested by the authors.

Early research showed intracerebroventricular (ICV) administration of N/OFQ to induce a reduction in locomotor activity in mice at comparatively high doses (1–10 nmol), which is a finding that was later corroborated in rats and with additional studies in mice at the same doses^10,19–21^. These effects were then theorized to occur indirectly via their actions on dopaminergic neurons. Indeed, N/OFQ inhibits dopamine transmission in striatal brain slices^22^. In another study, injection of N/OFQ resulted in regulation of motor performance in rats, with injection of a NOPr antagonist producing the opposite effect and leading to an increase in excitability of the motor cortex^23^. This motor behavior may be regulated by effects on cortical afferents produced by subcortical NOPr. Liu et al. demonstrated, in vitro, an inhibitory effect of a low dose of N/OFQ on the dopamine transporter, which inhibits dopaminergic activity^24^. They theorized this to be a potential mechanism for the decrease in locomotor activity seen in earlier studies. However, given the presence of NOPr on dopamine neurons and NOP mRNA in TH neurons, and the additional evidence of a decrease in motor cortex excitability, the NOPr system could also inhibit dopamine transmission via a direct impact on dopamine synthesis.

Di Giannuario et al. reported a reduction in morphine-induced dopamine release induced by treatment with N/OFQ in vivo^25^. On a similar stream, antagonism of NOPr has also been repeatedly shown to enhance dopamine transmission^26,27^. Marti et al. supported the in vitro evidence by demonstrating an inhibitory effect of N/OFQ administration on dopamine transmission in the striatum in vivo^26^. Marti et al. demonstrated these effects in a Parkinsonian model by showing improvements in Parkinsonian symptomatology following antagonism of NOPr in the nigrostriatal pathway, furthering the notion of an inhibitory effect of NOPr on dopamine transmission^27^. This evidence is further supported by additional preclinical investigations using Parkinsonian models^28–30^. Viaro et al^26^. demonstrated an attenuation of Parkinsonism in MPTP-treated mice with a NOPr antagonist, and a synergistic effect when this was employed with L-DOPA, indicating that the NOPr system was exerting its effects via dopamine transmission (also supported by Marti et al.^29^)^28,31^.

More recently, neuroprotective effects of NOPr downregulation on dopamine neurons were demonstrated by Arcuri et al.^32^. They observed a significantly greater (50%) amount of nigral dopamine neurons spared in mice following acute administration of MPTP. These findings led the authors to conclude that NOP-N/OFQ signaling contributes to dopamine neuron loss in Parkinson’s, speculated to be due to glutamate-mediated excitotoxic mechanisms, and provide support to previous findings^33,34^. Although the aforementioned evidence conveys a definite impact of the NOPr system on dopamine transmission, the exact mechanism by which this occurs is still unclear.

Ces et al.^33^ investigated NOPr signaling with pre-pulse inhibition (PPI), a validated model for schizophrenia and demonstrated an impairment of visual PPI with a NOPr agonist^35,36^. Authors also found that co-administration of haloperidol and the NOPr agonist attenuated PPI deficits, leading them to conclude that there is a functional cooperation between N/OFQ and dopamine. This evidence furthers the notion of the possibility of a role for NOPr signaling in schizophrenia.

### Involvement of the NOPr system in glutamate transmission

The glutamate hypothesis of schizophrenia is also well accepted, demonstrating hypofunction of the *N*-methyl-d-aspartate receptor (NMDAr), leading to a downregulation of glutamate^5^. In light of the significant impact of NOPr signaling on neurotransmission, glutamate transmission has also been studied. Nicol et al. showed decreased K^+^-evoked glutamate release in rat cerebrocortical, cerebellar, and brainstem slices in response to N/OFQ administration (see also Meis and Pape)^37–39^. Gompf et al. also showed N/OFQ to inhibit glutamate release in the retinohypothalamic tract and suprachiasmatic nucleus, and accorded this to be the result of presynaptic mechanisms by reducing Ca^2+^ (presynaptic release machinery)^40^. This was corroborated more recently by Kallupi et al., who demonstrated N/OFQ decreased glutamate release in the rat central amygdala^41^. Conversely, Marti et al. reported decreased glutamate release after NOPr antagonism in rats^27^. The difference in evidence may be reconciled by the consideration of the effects of NOPr on GABAergic signaling^42–47^.

Marti et al. reported a stimulatory effect of N/OFQ on nigral glutamate in vivo, and proposed this to be mediated via either dopaminergic or GABAergic mechanisms; this is because a GABA_A_ receptor antagonist was found to counter the effects of N/OFQ^48^. The GABAergic system is involved in the pathophysiology of psychosis, as has been demonstrated by post-mortem studies showing abnormal GABAergic interneurons (see review: Taylor and Tso and also Wassef et al.)^47,49^. Gavioli et al. showed NOPr signaling to be involved in anxiety through the GABA_A_ receptor, indicating through in vivo data the existence of effects of the NOPr system on GABAergic signaling^43^. Collectively, the data indicate a role of NOPr signaling in glutamate, as well as GABA transmission, with more evidence required to define the effects and exact mechanisms involved. A summary of these and additional findings is provided in Tables 1 and 2.

**Table 1:** NOPr in dopamine transmission

Experiment	Animal	Findings	Reference
N/OFQ ICV/haloperidol	Mice	↑ In locomotor activity (doses as low as 10 ng)/effect reversed with haloperidol	Florin et al.^107^
N/OFQ ICV	Mice and rats	↓ In locomotor activity	Reinscheid et al.^10^; Noble and Roques^20^; Devine et al.^19,108^; Rizzi et al.^21^; Narayanan et al.^109^; Chesnokova et al.^110^
NOPr knockout	Rats (crossed)	↑ Locomotor activity	Rizzi et al.^111^
N/OFQ ICV into SNr/UFP-101	Male Sprague-Dawley rats	↓ Motor activity/↑ motor activity with UFP-101	Marti et al.^23^
D2r knockout	Mice	↓ Motor facilitation by NOPr antagonists	Viaro et al.^18^
N/OFQ	Sprague-Dawley rats	↓ DAT activity/↓ GABA uptake	Liu et al.^24^
Dual in situ hybridization/6-OHDA	Male Sprague-Dawley rats/autoradiography	NOPr presence on DA neurons/NOP mRNA in TH neurons	Norton et al.^14^; Maidment et al.^112^
N/OFQ	Guinea pig and mouse striatal slices/rats/in vitro/primary culture/mice	↓ DA release (and cocaine-induced DA release)	Flau et al.^22^; Di Giannuario et al.^25^; Murphy et al.^113^; Zheng et al.^114^; Murphy and Maidment^46^; Murphy et al.^115^; Lutfy et al.^116^
UFP-101	Mice (wild type)	No effect on mesolimbic DA	Koizumi et al.^117^
Compound B	Mice	↑ DA release	Koizumi et al.^118^
N/OFQ microdialysis	Male Wistar rats	↑ DA release	Konya et al.^119^
N/OFQ perfused into SNr	Male Sprague-Dawley rats	↓ DA transmission in striatum	Marti et al.^26^
UFP-101	Male Sprague-Dawley rats	↓ Haloperidol-induced akinesia	Marti et al.^120^
N/OFQ after 6-OHDA and L-DOPA	Male Sprague-Dawley rats	↓ L-DOPA-induced dyskinesia (with N/OFQ)	Marti et al.^121^
6-OHDA or MPTP/J-113397/UFP-101/Compound 24	Male Sprague-Dawley rats/mice	↓ Parkinsonian symptoms	Marti et al.^27^; Viaro et al.^29^; Volta et al.^30^
6-OHDA / Trap-101/L-dopa	Sprague-Dawley rats/mice	↓ Parkinsonian symptoms	Marti et al.^28^; Viaro et al.^31^
N/OFQ	Male Sprague-Dawley rats	↓ TH phosphorylation	Olianas et al.^17^
6-OHDA	Male Sprague-Dawley rats	↓ N/OFQ, NOPr mRNA in caudate putamen	Di Benedetto et al.^15^
CSF measurements in PD patients	N/A	↑ N/OFQ in striatum and substantia nigra	Marti et al.^122^
Ro64-6198	Mice	↓ Visual PPI	Ces et al.^35^
MPTP	Mice	↑ Nigral N/OFQ mRNA	Gouty et al.^123^
NOPr or N/OFQ knockout/MPTP/methamphetamine	Mice	↑ Sparing of nigral DA neurons (and striatal)	Brown et al.^33^; Arcuri et al.^32^; Sakoori and Murphy^34^

*ICV* intracerebroventricular injection, *6-OHDA* 6-hydroxydopamine lesions, *J-113397* NOPr antagonist, *UFP-101* NOPr antagonist with partial agonist properties,* MPT*P 1-methyl-4-phenyl-1,2,3,6-tetrahydropyridine, *Trap-101* NOPr antagonist, *compound 24 and compound B* NOPr antagonists, *Ro64-6198* NOPr agonist, *DAT* dopamine transporter, *TH* tyrosine hydroxylase, *DA* dopamine, *PPI* prepulse inhibition, *D2r* dopamine D2 receptor subtype, *SNr* substantia nigra pars reticulata

**Table 2:** NOPr in glutamate transmission

Experiment	Animal	Findings	Reference
N/OFQ	Female Wistar rats	↓ K^+^-evoked glutamate release in cerebrocortical, cerebellar, and brainstem slices	Nicol et al.^37,38^
N/OFQ	Long Evans rats	↓ Non-NMDA EPSC	Meis and Pape^39^
NOPr knockout	Mice	↑ NMDAr function	Mamiya et al.^124^
J-113397 intraperitoneally	Mice	↓ glutamate release	Mabrouk et al.^42^; Marti et al.^45^
N/OFQ microdialysis/ NOPr antagonist	Male Sprague-Dawley rats	↑ Nigral glutamate release (countered by antagonist)	Marti et al.^48^
UFP-101	Male Sprague-Dawley rats	↓ Nigral glutamate release (normalized)	Marti et al.^120^
N/OFQ	Male Sprague-Dawley rats	↓ Glutamate-mediated EPSC	Gompf et al.^40^
J-113397/UFP-101	Male Sprague-Dawley rats	↓ Glutamate release	Marti et al.^27^
N/OFQ	Male Wistar rats	↓ Glutamatergic receptor-mediated EPSPs	Kallupi et al.^41^

*EPSC/EPSP* excitatory post-synaptic current/potential, *NOPr antagonist* [Nphe^1^]nociceptin/orphanin FQ(1–13)NH_2_

Together, the evidence of NOPr involvement in dopamine and glutamate signaling, given the localization patterns and modulatory roles, suggests considerable potential for NOPr signaling in the pathophysiology of schizophrenia.

### Involvement of the NOPr system in the HPA axis

Patients with schizophrenia present with an increased vulnerability to stress, which is thought to be a result of HPA axis dysregulation^6^. The NOPr system may be a critical mediator in the stress response via the HPA axis through its effects on adrenocorticotropic hormone and corticosterone. The collective findings are summarized in Table 3. N/OFQ increased corticosterone and adrenocorticotropic hormone in non-stressed rats and in mildly stressed rats, indicating its ability to activate the HPA axis^50^. In a similar manner, stress decreased N/OFQ content in the basal forebrain^51^. Leggett et al. also observed increased plasma adrenocorticotropic hormone and corticotropin-releasing factor mRNA in the paraventricular nucleus, known to be instrumental in HPA axis activity, in response to N/OFQ, leading the authors to conclude that N/OFQ mediates HPA axis activation^52^. Limbic system involvement is also evident, as acute restraint stress increased N/OFQ expression in hippocampal subfields and was associated with concentration of glucocorticoids^53^. However, HPA axis activity effects appear to occur through additive actions in multiple brain regions, since ICV injection of N/OFQ also resulted in elevated corticosterone levels^54^.

**Table 3:** NOPr in stress modulation

Experiment	Animal	Findings	Reference
N/OFQ knockout	Mice	↑CORT	Jenck et al.^55^
N/OFQ knockout/N/OFQ ICV	Mice	↓ Adaptability to repeated stress/↑ adaptability to stress (with N/OFQ ICV)	Koster et al.^56^; Griebel et al.^125^
N/OFQ	Male Sprague-Dawley rats	↑CORT, ACTH in unstressed rats and mildly stressed rats	Devine et al.^50^
N/OFQ ICV	Mice	↓CORT	Le Cudennec et al.^57^
N/OFQ ICV	Male long Evans rats	↑ CORT	Green et al.^54^
N/OFQ subcutaneously and injections	Mice	↑CORT (only injections do this—thus reconsider methodology and environmental stressors)	Prince-Zullig et al.^62^
N/OFQ ICV	Male Sprague-Dawley rats	↑plasma ACTH, CRF mRNA in PVN	Leggett et al.^52^
Acute restraint stress+NOPr antagonism	Male Sprague-Dawley rats	↑ Activation of HPA axis in nadir phase (not in peak phase)	Leggett et al.^126^
LPS+NOPr antagonism	Male Sprague-Dawley rats	↓ Activation of HPA axis	Leggett et al.^127^
Social defeat stress	Male/female Long Evans rats	↑ NOPr mRNA in PVN	Green and Devine^58^
JTC-801+acute restraint	Male Sprague-Dawley rats	↑ HPA axis response/↓ NOPr gene expression in hypothalamus	Delaney et al.^128^
Acute restraint stress	Male Sprague-Dawley rats	↓N/OFQ in basal forebrain	Devine et al.^51^
Social stress	Mice	↑ NOPr mRNA, N/OFQ precursor mRNA	Reiss et al.^59^
Acute restraint stress	Male Wistar rats	↑ N/OFQ in HPC subfields	Nativio et al.^53^
Acute restraint stress	Male Wistar rats	↑ N/OFQ	Ciccocioppo et al.^60^
Social defeat stress	Male Wistar rats	↑ N/OFQ mRNA in NAcc shell	Der-Avakian et al.^61^

*LPS* lipopolysaccharide (physiological stress), *JTC-801* NOPr antagonist with partial agonist properties, *CORT* corticosterone, *ACTH* adrenocorticotropic hormone, *CRF* corticotropin-releasing factor, *PVN* paraventricular nucleus, *HPA* *axis* hypothalamic–pituitary-adrenal axis, *HPC* hippocampus, *NAcc* nucleus accumbens

Genetic knockout of N/OFQ reduced the adaptability of mice to stress, but resulted in an elevated level of plasma corticosterone, demonstrating that the effect of NOPr on the HPA axis may be inhibitory^55^. In another study, N/OFQ knockout mice had impaired adaptation to stress, furthering the theory of NOPr involvement in stress adaptability^56^. In this study, exposure to repeated stress by way of a forced-swim test failed to produce adaptability in knockout mice, while an increase in anxiety-like behavior was also noted.

Similarly, Le Cudennec et al. showed N/OFQ to decrease corticosterone levels following stress, indicating anti-stress effects of this neurotransmitter and also an inhibitory effect on the HPA axis^57^. These differential findings may arise due to species differences, but may also indicate a dynamic role of NOPr modulation of stress reactivity. Social stress (in different forms) increased N/OFQ and NOPr mRNA in the hippocampus, central amygdala, paraventricular nucleus, and in the nucleus accumbens shell^58–61^.

Differential results do exist, such as those obtained by Prince-Zullig et al.^62^ They reported no difference in basal corticosterone levels between N/OFQ knockout mice and wild-type controls, in direct contrast with those of Koster et al.^56^ Additionally, they found N/OFQ administration to have no significant impact on corticosterone levels compared to saline-injected controls, contradicting prior evidence and suggesting a role of environmental stressors or the injection procedure itself in producing these elevated responses. Such effects have also been noted previously (see review: Gavioli et al.^63^). These differences may arise from the NOPr system’s significant involvement in pain, as one study showed a lack of HPA activation in a neuropathic pain model, thus implicating pain in HPA axis activation^64^.

We also note that the NOPr system plays a role in the production of cytokines, with several studies demonstrating its peripheral impact on the immune system (see review: Bodera et al.)^65–67^. This could also account for HPA axis activation. Nonetheless, the accumulation of this evidence thus far aligns itself with the findings of a dysregulated HPA axis in schizophrenia patients^6^, given the apparently modulatory role of NOPr on the HPA axis.

### Involvement of the NOPr system in cognition

Cognitive deficits are a prevalent finding in the schizophrenia population, with deficits in working memory being commonplace^2^. The NOPr system has been shown to play a role in cognition, based on evidence from preclinical studies. These findings are summarized in Table 4. Initial evidence demonstrated spatial learning deficits after N/OFQ injection into the rat hippocampus, and blocking of these effects by NOPr antagonism^68–70^. Higgins et al. also observed improved performance in N/OFQ knockout rats, with dose-dependent reductions in swim speed, demonstrating effects on locomotion^70^. Similarly, Sandin et al. demonstrated a dose-dependent biphasic effect of N/OFQ on spatial learning, with low doses improving learning and higher doses impairing it^71^. In contrast, Kuzmin et al. replicated these findings in mice with ICV administration of N/OFQ and observed no biphasic effect, suggesting potential species differences^72^. Questions regarding the mechanism of NOPr signaling effects on cognition do exist, as Koster et al. demonstrated N/OFQ knockout mice to have no difference in spatial learning compared to controls^56^.

**Table 4:** NOPr in cognition

Experiment	Animal	Findings	Reference
N/OFQ intrahippocampally	Male Sprague-Dawley rats	↓ Spatial learning in MWM	Sandin et al.^68^; Redrobe et al.^69^
N/OFQ knockout	Mice	No effect on spatial learning	Koster et al.^56^
Retro-nociceptin methylester	Mice	↑ Learning ability	Jinsmaa et al.^129^
N/OFQ	Mice	↓ Latent learning	Noda^130^
NOPr knockout	Mice	↑ Spatial learning (↓ DA in frontal cortex in knockout mice)	Mamiya et al.^131^
Ro64-6198/N/OFQ knockout	Mice/Lister hooded rats	↓ Spatial learning in MWM/ ↓ LTP	Higgins et al.^70^
N/OFQ intrahippocampally	Male Sprague-Dawley rats	↓ Spatial learning at high doses/↑ spatial learning at low doses (biphasic effect)	Sandin et al.^71^
N/OFQ ICV/ Prepro N/OFQ knockout	Mice	↓ Spatial learning in MWM/ ↑ learning in knockout mice	Kuzmin et al.^72^
N/OFQ ICV	Mice	↓ Working memory in passive avoidance task	Hiramatsu and Inoue^74^; Liu et al.^132^
NOPr knockout	Mice	↑ Learning and memory (with novel KUROBOX apparatus)	Nagai et al.^133^
NOPr knockout	Mice	↑ Working memory in passive avoidance task	Mamiya et al.^73^
NOPr knockout	Mice	↑ Learning ability and memory	Noda et al.^134^
NOPr knockout	Mice	↑ Working/spatial memory in MWM, passive avoidance task/ ↑ LTP	Manabe et al.^76^; Taverna et al.^135^
N/OFQ (tetanic stimulation)	Mice	↓ LTP (hippocampal CA1 region)	Bongsebandhu-phubakdi and Manabe^78^
N/OFQ	Male Sprague-Dawley rats / Mice	↓ LTP in HPC/↓NMDAr-mediated EPSC / ↓LTD	Yu et al.^82^; Yu and Xie^75^; Wei and Xie^77^
N/OFQ intrahippocampally	Mice	↓ Memory impairment	Miwa et al.^136^
Ro64-6198/mecamylamine	Mice	↓ Recognition memory in object recognition task	Reiss et al.^79^
N/OFQ ICV or Ro64-6198/MK-801	Mice	↓ Recognition memory in object recognition task /↓ long-term memory formation (administered together)	Goeldner et al.^80^
N/OFQ ICV	Male Wistar rats	↓ ACh release in striatum	Itoh et al.^83^
N/OFQ	Rats—in vitro	↓ ACh efflux in cortical and hippocampal slices	Cavallini et al.^84^
NOPr knockout	Mice	↑ ACh in hippocampus (and ↑ hippocampal theta rhythm)	Uezu et al.^85^
N/OFQ ICV	Male Sprague-Dawley rats	↓ Mecamylamine impairment at low doses / memory impairment at high doses	Hiramatsu et al.^86^

*MWM* Morris water maze, *LTP* long-term potentiation, *LTD* long-term depression, *Retro-nociceptin methylester* NOPr antagonist, *ACh* acetylcholine, *Mecamylamine* nicotinic receptor antagonist

Moreover, working memory impairments have been noted via insufficiencies in passive avoidance in animals following NOPr activation through administration of agonists or N/OFQ^70,73,74^. NOPr activation impaired long-term memory formation as measured through recognition memory. The mechanism for this is potentially via the suppression of glutamatergic function at the NMDA receptor^75–78^. Reiss et al. demonstrated selective impairment of recognition memory in mice following co-administration of a NOPr agonist and NMDA receptor antagonist, further demonstrating NOPr system modulation of memory formation via glutamatergic receptors^79^. These findings have since found additional support with deficits in recognition memory and fear learning in mice following increases in NOPr activity mediated via suppression of glutamate transmission^80,81^. Furthermore, a negative impact of NOPr signaling on long-term potentiation (LTP) in the hippocampus has also been observed, as NOPr-deficient mice had improved LTP, gauged through NOPr and N/OFQ gene expression in the hippocampus^70,75,76,82^.

Acetylcholine (ACh) signaling is posited to play a role in the cognitive deficits observed in schizophrenia^7^, and the NOPr system is also implicated with this neurotransmitter. Initial in vivo evidence showed N/OFQ to decrease ACh release in the striatum in rats^83^. This was later corroborated with similar evidence in cortical and hippocampal regions, thus further demonstrating effects of the NOPr system on cognition^84^. Uezu et al. reported specific findings in the hippocampus, with knockout mice having greater amounts of ACh, leading the authors to speculate an impact of NOPr signaling on memory function^85^. Findings by Hiramatsu et al. indicate a dose-dependent effect of N/OFQ on ACh signaling, as they found high doses to decrease it, while lower doses countered antagonist-induced ACh signaling decrease^86^. Additional research is necessary in order to further elucidate the mechanisms by which these effects occur.

Collectively, these results confirm an involvement of NOPr signaling on cognition including working memory deficits, spatial working memory deficits, and impairments in LTP. Dopaminergic dysfunction, glutamatergic hypofunction, and effects on cholinergic transmission have been outlined as mechanisms that may underlie these deficits^87–89^, and hence, aberrant NOPr signaling may play a crucial role in the cognitive deficits of schizophrenia.

### Involvement of the NOPr system in reward modulation

Deficits in reward processing and motivation are a common finding in schizophrenia^3^. Through investigations into the rewarding properties of drugs of abuse, the NOPr system could be involved in reward modulation, particularly since a moderate-to-high concentration of NOPr in regions associated with reward is observed, including the ventral tegmental area, medial prefrontal cortex, amygdala, and the bed nucleus of the stria terminalis^11^. The findings are summarized in Table 5. Conditioned place preference (CPP) is a valid method for the study of motivational effects and reward in different paradigms^90^. Thus, studies with NOPr signaling in CPP can aid understanding of the role of the NOPr system in reward. Treatment with N/OFQ resulted in an inhibition of reward salience, as measured via CPP, and these findings were replicated with multiple drugs of abuse, including morphine, cocaine, and amphetamines^91–95^. Similarly, NOPr antagonism or knockout results in an increase in CPP with drugs of abuse^96–98^. However, the literature is still inconsistent as an increase in CPP has also been observed with NOPr agonism^98,99^. Endogenous N/OFQ does not have any reinforcing effects, indicating that it in itself does not have any effects on CPP^98^. Generally, activation of the NOPr system could be involved in negative reinforcement, as agonism has been shown to decrease self-administration of ethanol^100^.

**Table 5:** NOPr in reward modulation

Experiment	Animal	Findings	Reference
Ro65-6570/N/OFQ	Male Wistar rats	↑ CPP with cocaine/↓ CPP with cocaine	Kotlinska et al.^99^
NOPr knockout	Mice	↑ CPP with cocaine	Marquez et al.^96^
N/OFQ ICV	Male Sprague-Dawley rats	↓ Cocaine-induced DA release in NAcc	Vazquez-Derose et al.^92^
Ro65-6570/J-113397	Male Sprague-Dawley rats	↑ CPP/↓ CPP with antagonist (with opioids)	Rutten et al.^98^
N/OFQ ICV	Male Wistar rats/male Sprague-Dawley rats	↓ CPP with morphine/↓ sensitization to morphine (using agonists)	Ciccocioppo et al.^93^; Murphy et al.^137^; Kotlinska et al.^138^
J-113397	Male Sprague-Dawley rats	↑ CPP with morphine	Rutten et al.^91^
N/OFQ ICV	Mice	↓ CPP with morphine and cocaine	Sakoori and Murphy^94^
Ro64-6198	Male Wistar rats	↓ Ethanol self-administration	Kuzmin et al.^100^
N/OFQ knockout	Mice	↑ CPP with methamphetamine and ethanol	Sakoori and Murphy^97^
N/OFQ ICV	Male Sprague-Dawley rats	↓ CPP with methamphetamine	Zhao et al.^139^
N/OFQ ICV	Male Wistar rats	↓ CPP with amphetamine	Kotlinska et al.^95^
NOPr knockout	Rats (Wistar controls)	↓ Self-administration of cocaine, heroin, ethanol/no difference on saccharin self-administration compared to controls	Kallupi et al.^102^
Cebranopadol	Male Wistar rats	↓ Self-administration of cocaine/no effect on sweetened condensed milk self-administration	de Guglielmo et al.^103^
N/OFQ ICV	Mice	↑ Licking for sweet solutions	Mendez et al.^101^

*Ro65-6570* NOPr agonist (w/out motivational properties—unlike Ro64-6198), *CPP* conditioned place preference, *Cebranopadol* NOPr full agonist (also full agonist of mu, partial agonist of kappa and delta opioid receptors)

This discrepancy is highlighted by more recent findings regarding the NOPr system in reward and motivation. ICV administration of N/OFQ increased bouts of licking for sucrose (a sweet solution) in mice, which led to the suggestion that activation of this system increases the motivation associated with appetite^101^. Conversely, NOPr knockout rats in another study did not differ from wild-type controls in their preference for saccharin, although these rats did have a significantly reduced proclivity for self-administration of cocaine, heroin, and alcohol^102^. A similar finding was observed in a comparison between cocaine and sweetened condensed milk^103^. These results suggest a role for the NOPr system in drug reward specifically, but are also inconsistent with previous findings of a role in motivation in general. These differences could be a result of inherent species differences as well as a difference in methodology (i.e., licking microstructure analysis versus fixed-ratio self-administration).

Another recent study demonstrated negative correlations between reward learning and N/OFQ peptide mRNA levels in the cingulate gyrus and with NOPr mRNA levels in the ventral tegmental area^61^. Overall, the results align with the reward system disruptions noted in schizophrenia, in which the existence of deficits is well supported by the literature^3^. The NOPr system may thus play a role in these deficits, further potentiating its involvement in the pathophysiology of schizophrenia.

### Conclusion and future directions

In summary, the literature indicates a role of the NOPr system in dopamine and glutamate regulation, with NOPr activation generally decreasing dopamine and glutamate transmission, although this requires further elucidation. Activation of NOPr is also associated with HPA axis regulation, implicating a role for it in the modulation of stress. Cognition is generally negatively impacted with NOPr activation. While reports of the system’s impact on the reward system are mixed, they nonetheless point to the existence of an effect. Indeed, the NOPr system has potential in other psychiatric illnesses, such as depression, in which antagonism is demonstrated to have promising effectiveness^104^.

Due to the multi-faceted effects of the NOPr system in the brain, how exactly it may be altered in psychosis remains to be elucidated. This preclinical and in vitro evidence presented in conjunction with the well-replicated findings in schizophrenia clearly implicate a plausible contribution of the NOPr system in the pathophysiology of schizophrenia^89,105^. The literature we have presented in this review indicates the paucity in this field and thus highlights the need for further research. The development and validation of the novel PET tracer [^11^C]NOP-1A now makes this possible to investigate in clinical populations^13,106^.

In conclusion, we present here a novel approach to a complex neuropsychiatric illness and demonstrate that the literature suggests a potential role of the NOPr system in schizophrenia, with ramifications in the development of better treatment and interventions, and possibly even prevention.
